# Virulence of *Naegleria fowleri* isolates varies significantly in the mouse model of primary amoebic meningoencephalitis

**DOI:** 10.1101/2025.05.20.655168

**Published:** 2025-05-20

**Authors:** A. Cassiopeia Russell, Erica Schiff, Joseph Dainis, Håkon Jones, Christopher A. Rice, Dennis E. Kyle

**Affiliations:** University of Georgia, Athens, Georgia, USA

**Keywords:** *Naegleria fowleri*, virulence, pathogenicity, primary amoebic meningoencephalitis

## Abstract

*Naegleria fowleri* is a small free-living amoeba that causes an acute, fatal disease called primary amoebic meningoencephalitis (PAM). One persisting question is why few people succumb to disease when so many are potentially exposed. We tested the hypothesis that *N. fowleri* isolates vary in virulence and in the minimum infectious dose required to induce disease by using a mouse model of PAM and intranasally inoculating dilutions of five clinical isolates of *N. fowleri*. Results showed significant differences in onset of severe disease and mortality rates between isolates. Remarkably, for isolate V596, 100 amoebae produced 100% mortality within 5 days. In contrast, higher numbers of amoebae were required for other isolates and mice survived for >2 weeks. Concurrently, we developed an *in vitro* virulence assay by comparing feeding rates between amoebae isolates seeded onto Vero cells. We observed a positive correlation between cytopathic effects *in vitro* and virulence *in vivo*.

## Article Summary Line

Isolates of *Naegleria fowleri* vary in virulence, which results in significant differences in minimal infectious concentrations of amoebae that cause primary amoebic meningoencephalitis.

Small, thermotolerant free-living amoebae are ubiquitous in soil and freshwater in the environment, although several pathogenic free-living amoebae are known to cause serious diseases in humans.(1) *Naegleria fowleri*, colloquially known as the brain-eadng amoeba, is the causadve agent of primary amoebic meningoencephalids (PAM). Humans become infected while pardcipadng in freshwater acdvides or performing nasal abludons when water containing the amoebae enters the nasal passages; the amoebae then traverse the olfactory epithelium and migrate to the frontal lobes of the brain where they cause significant pathology. Meningids symptoms usually begin within a week afer warm water exposure, and severe complicadons followed by death occur in the next 1–18 days. Although PAM reportedly is a rare disease, it unfortunately has a >98% case fatality rate with no survivors in the US since 2016.(2) There are several lines of evidence that suggest PAM is under-reported in warm climates around the world. Firstly, in Pakistan, where ritualisdc nasal abludon is a common pracdce, large outbreaks have been documented recently once surveillance was insdtuted.(3, 4) In addidon, the early signs and symptoms of PAM are similar to meningiddes caused by viral or bacterial pathogens, therefore most PAM cases are misdiagnosed or detected late in the infecdon, or postmortem.(1) Lastly, recent studies reported high rates of seroposidvity to *N. fowleri* in people from several regions of Mexico.(5) These data suggest high rates of environmental exposure to *N. fowleri*, which contrasts sharply with the low rates of infecdon and disease in humans.

Quite ofen the source of infecdon with *N. fowleri* is linked to a site where hundreds of people presumably had similar exposure to amoebae in the same dme frame as the individual that contracted the deadly infecdon. These include lakes,(6, 7) hot spring-fed pools,(8) whitewater recreadon areas,(9) inadequately treated potable water (e.g., non-sterile tap water used in ned-pots),(10) and ardficial water sports venues,(11) to name a few. Since the numbers of documented PAM cases are low and exposure to amoebae in warm water is high, a major quesdon is why some people get infected with *N. fowleri* and develop PAM, whereas others do not. There are many possible hypotheses for low rates of infecdon and disease; these include host and parasite factors which are difficult to study with the currently available tools.

In this study we hypothesized that *N. fowleri* isolates vary in virulence and that these differences affect the minimal infecdous concentradons of amoebae required to inidate a fulminant infecdon. To test our hypothesis, we evaluated virulence of five clinical isolates of *N. fowleri* in the mouse model of PAM. In parallel, we also developed an *in vitro* cytopathogenicity model that demonstrated posidve correladon between *in vivo* and *in vitro* virulence phenotypes.

## Methods

### *N. fowleri* isolates

All isolates of *N. fowleri* used in this study were collected from clinical cases of PAM (Table 1). ATCC 30215 (Nf69) was purchased from the American Type Culture Collecdon (ATCC), and the remaining isolates were generously provided by Dr. Ibne Ali, US Centers for Disease Control. The dates of isoladon for the cases ranged from 1966 – 2011 and most of the padents were male (10 of 13). The isolates included examples of three genotypes, although genotypes I and III were the best represented groups in this collecdon. All isolates were roudnely maintained in axenic culture in Nelson’s complete media (NCM) as previously described (12).

### *In vivo* virulence

**Figure 1.**
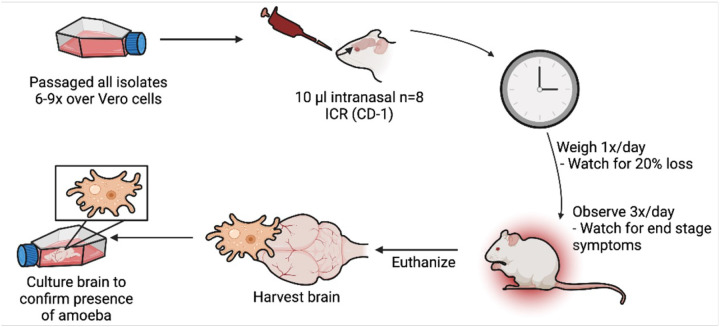
Schemadc overview of methods used for *in vivo* virulence studies. *N. fowleri* strains were first passaged over Vero cells at least 6 dmes. Amoebae were washed and diluted to the desired amoeba number in 10 μL and then dosed intranasally into mice (n=8 per group). Animals were observed muldple dmes per day and euthanized when symptoms of disease were observed. Brains of mice were harvested and inoculated into culture media to confirm the presence of amoebae. (Created with BioRender.com.)

In preparadon for an *in vivo* experiment, first the amoebae from axenic cultures were centrifuged and washed 2 dmes in 1X phosphate buffered saline (PBS) before adding to flasks containing Vero cell (green monkey kidney cells; E6; ATCC CRL-1586) monolayers (Figure 1). Vero cells were cultured and sub-cultured as previously described (13). The amoebae-host cell cocultures were monitored daily and subculture to a new passage was made once most (≥85%) of the Vero cells had been destroyed by the amoebae. We passaged the amoebae ≥ 6 dmes on Vero cells to “prime” them for infecdon and then collected and concentrated amoebae by centrifugadon before preparing stock concentradons containing 1000, 10,000, or 50,000 trophozoites in 100 μl of PBS. Then 10 μl of PBS containing amoebae was then inoculated into a single nare of an anesthedzed female 3–4-week-old ICR (CD-1) mouse, ranging from 17–26.5g in weight. Either 5 or 8 mice were infected per isolate-concentradon study, whereas n=5 mice were used as uninfected control animals.

Daily weight monitoring was performed as well as thrice daily inspecdons for symptoms of PAM, including piloerecdon, orbital dghtening, arched spine, ataxia, increased respiratory effort and seizures. Animals displaying ≥20% weight loss or end-stage symptoms were euthanized per approved animal use protocols to midgate pain or suffering. Brains were dissected from each animal; each brain was then placed into a flask containing 10 ml of NCM containing 10 U/ml of penicillin and streptomycin, and cultured for up to 7 days to microscopically confirm presence of amoebae. All surviving mice were euthanized on day 35 post infecdon to conclude the study.

An addidonal follow-up animal study was performed as described above to idendfy a minimum infecdous dose for isolates that caused acute mortality at the lowest doses of 100 and 1000 amoebae. We udlized n=5 mice per concentradon of isolate, with n=3 uninfected control mice. Isolate V596 was inoculated at concentradons of amoebae of 1000, 100, 75, 50, 25, or 10 per mouse. Isolate Villa Jose was inoculated at concentradons of amoebae of 5000, 2500, 1000, 500, 250, or 100 per mouse. Data from these studies were used to determine the 50% lethal concentradons (LC_50_s) of *N. fowleri* amoebae required to induce a fulminant infecdon in mice. Survival data and LC_50_s were calculated by using GraphPad Prism sofware (version 10, GraphPad, La Jolla, CA, USA). All animal studies were reviewed and approved by the University of Georgia Insdtudonal Animal Care and Use Committee 105 (Protocol A202 03–026). All animal studies were conducted according to the Guide for the Care and Use of Laboratory Animals.

### *In vitro* virulence

For the *in vitro* virulence assay, we used the culture methods described above and first passaged each isolate 5 dmes over Vero cell monolayers in culture flasks. We then concentrated the cells by centrifugadon and split the amoebae into three flasks for biological replicates. To prepare for the assay, 24 hours before the 5^th^ passage of amoebae finished feeding upon monolayers, we added 25,000 Vero cells/well in a μClear black Cellstar 96-well microplate (Greiner Bio-One, Kremsmunster, Austria) and incubated for two days at 37°C undl the monolayer reached 80–90% confluency was formed. For inidal opdmizadon, test plates contained a diludon series of 100 to 50,000 amoebae and dme checks were performed at 3 h, 6 h, 12 h, and 24 h to determine ideal amoebae seeding density and dme for the cytopathogenicity assay. We determined that a maximal and minimal concentradon of 10,000 and 625 amoebae, respecdvely, and an incubadon dme of 24h allowed for visible clearance of monolayers that could be measured with high content imaging. For the assay, passage 5 amoebae were harvested and serial diludons of amoebae, from 10,000 to 625, were prepared and each concentradon was added in quadruplicate wells containing confluent Vero cells.

Afer 24 h co-incubadon, media was carefully aspirated to avoid disturbing adherent cells, and 100 μl of 4% paraformaldehyde (PFA) in phosphate-buffered saline (PBS) was added with an incubadon period of 15 min in the dark at room temperature. Fixadve was removed and a wash with 100 μl PBS was performed prior to adding 100 μl of 10 μg/ml Hoechst 33342. Plates were incubated at room temperature in the dark for 45 min prior to stain removal and a final PBS wash. PBS (100 μl) was added to each well and high content imaging was conducted on an ImageXpress Micro Confocal HCI system (Molecular Devices, San Jose, CA, USA) to idendfy nuclei stained with Hoechst 33342, quandfy host cell count, and exclude smaller amoebae nuclei from cell totals. Final imaging parameters were set to count Vero cell nuclei, and the assay endpoint was the number of Vero cell nuclei remaining afer 24 h. Vero-cell-only control wells were compared to amoeba serial diludon wells and percentage feeding rate comparisons were prepared using GraphPad Prism sofware (version 10, GraphPad, La Jolla, CA, USA).

## Results

Our first aim was to directly compare levels of virulence of different clinical isolates of *N. fowleri* in the ICR (CD-1) mouse model of PAM. For these studies we used five clinical isolates (Table 1). Given the lack of informadon regarding prior passage number and culture condidons of the isolates, we devised a scheme to allow direct comparison of these isolates. Previous studies suggest that passaging amoebae over mammalian cells can enhance pathogenicity in mouse infecdons.(14) Therefore, we standardized the preparadon of amoebae for animal studies by passaging them 6–9 dmes over Vero cells to induce acdve feeding before preparing diludons to be used to inidate PAM infecdon in mice (Figure 1).

**Figure 2.**
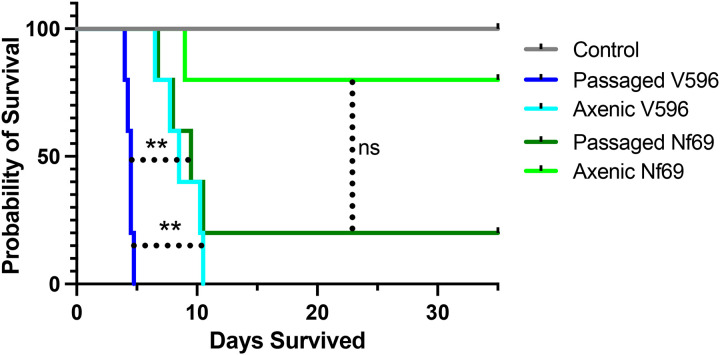
Survival curves for mice infected with lowly (Nf69) and highly (V596) virulent *N. fowleri* isolates show significant differences in survival. Differences in survival are also noted for passaged vs axenically cultured isolates (**p < 0.01; ns = not significant).

The udlity of this approach was demonstrated with a comparison of virulence in mice that were inoculated with amoebae from standard axenic culture (Nelson’s media) versus amoebae passaged over Vero cells. As shown in Figure 2, both the V596 and Nf69 strains of *N. fowleri* that were fed on Vero cells were significantly more virulent in mice than amoebae grown in axenic media. In addidon, the enhanced virulence phenotype was observed with the most virulent strain (V596) as well as with the less virulent Nf69 strain.

**Figure 3.**
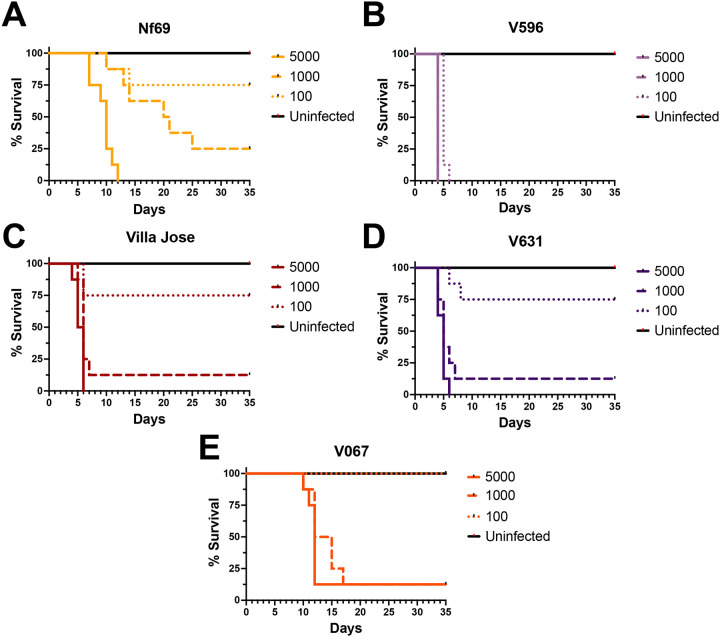
*N. fowleri* isolates exhibit varying levels of virulence in the mouse model of Primary Amoebic Meningoencephalids (PAM). Amoeba numbers inoculated intranasally for each strain were 100, 1000, and 5000. The survival dmes to euthanasia due to severe symptoms of disease are shown for Nf69 (A), V596 (B), Villa Jose (C), V631 (D), and V067 (E), respectively.

Afer validadng the method, next we assessed virulence of five isolates of *N. fowleri* in the mouse model of PAM. Interesdngly, these strains demonstrated significantly different levels of virulence *in vivo*, with mean survival dmes ranging from 4 days to ≥35 days (Figure 3). Nf69, a strain we have used as a lab standard for drug discovery studies,(15, 16) was moderately virulent. All mice inoculated with 5000 Nf69 amoebae succumbed to infecdon, yet only 75% and 25% died afer infecdng mice with 1000 or 100 amoebae, respecdvely. In contrast, V596 was the most virulent isolate tested. We observed 100% mortality with a mean survival dme of 4–5 days, even with as few as 100 amoebae inoculated into a single nare of a mouse. V631, Villa Jose, and V067 produced mean survival dmes ranging from 6 to 14 days with inocula of 1000 amoebae per mouse. Interesdngly, 25% of the mice succumbed to disease that were infected with 100 Villa Jose or V631; all mice inoculated with 100 V067 amoebae survived for 35 days. Differences in gross pathology of brains were observed in mice infected with V596 versus less virulent N. fowleri strains (Appendix Figure 1). These data demonstrate significant differences in virulence of *N. fowleri* strains.

**Figure 4.**
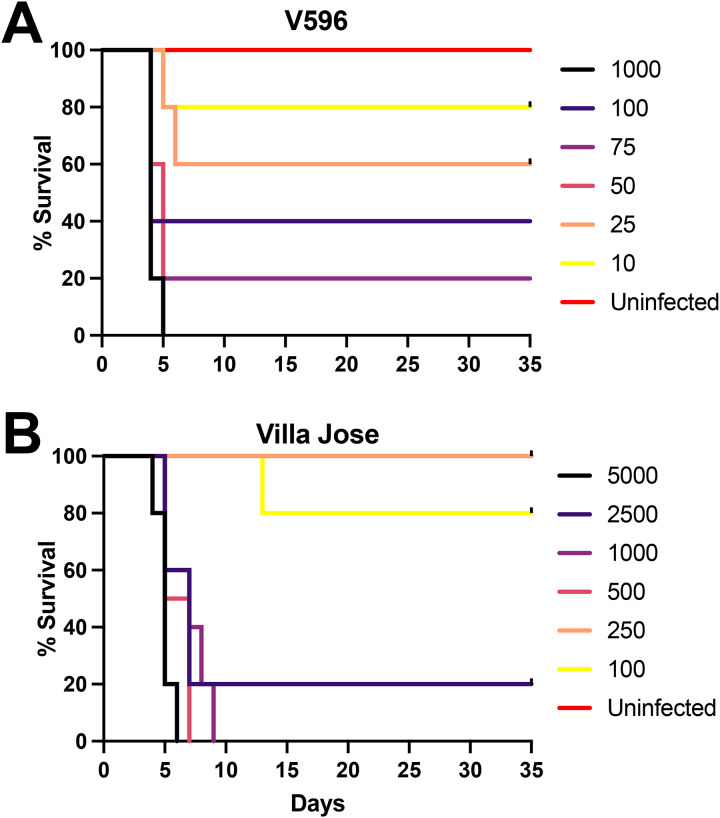
A follow-up *in* vivo study was conducted to idendfy the minimal infecdous concentradons of V596 (A) and Villa Jose (B) strains of *N. fowleri* amoebae that caused 100% mortality in the mouse model of PAM. V596 was found to be the most virulent strain, requiring only 10 amoebae to cause death and an esdmated 50% lethal concentradon of ~40 amoebae. The minimal 100% lethal concentradon of Villa Jose was 1000 amoebae, with an esdmated LC_50_ of 325 amoebae.

In follow up mouse studies we conducted a comparison study to assess the 50% lethal concentradons of V596 and Villa Jose amoebae to induce a fatal PAM disease (Figure 4). For these studies we inoculated mice with to 10, 25, 50, 75, 100, and 1000 amoebae and assessed mouse survival dmes. These studies confirmed that V596 is the most virulent isolate tested, with deaths observed in <6 days with as few as 10 – 25 amoebae. The survival curves for Villa Jose also were reproducible with inocula of ≤500 amoebae producing fulminant infecdons. Based upon these studies, the esdmated 50% lethal concentradons (LC50s) are ~40 and ~325 amoebae for V596 and Villa Jose, respecdvely. In addidon to providing quandtadve esdmates of virulence differences between isolates, the reproducibility of survival data demonstrates the udlity of the standardized method of feeding amoebae over Vero cells prior to conducdng *in vivo* studies.

### *In vitro* virulence assay

For addidonal studies on mechanisms of virulence in *N. fowleri*, it would be advantageous to have a facile, quandtadve *in vitro* assay for assessing virulence phenotype:genotype associadons in a high-throughput assay. During the conduct of the Vero cell passages for the *in vivo* studies, we observed that isolates cleared the Vero cell monolayers at different rates. Some isolates would destroy the complete Vero cell monolayer within 24 h, whereas others would take several days to clear the mammalian cells. Thus, we hypothesized that the rate at which *N. fowleri* isolates feed on Vero cells could serve as a surrogate endpoint for virulence, with the addidonal prospect of serving as a surrogate of *in vivo* virulence.

**Figure 5.**
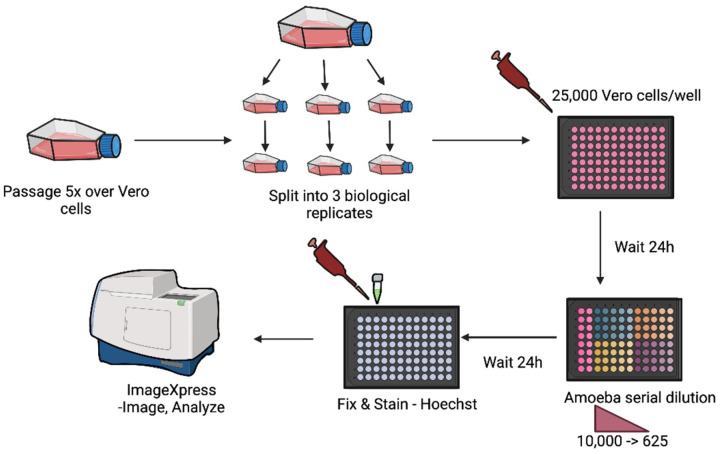
Schemadc of methods used for the in vitro virulence assay. *N. fowleri* trophozoites were passaged 5 dmes over Vero cells, washed, and then split into three biological replicates. Vero cells were added to a 96 well plate and 24 hr later serial diludons of 625- to 10,000-amoebae were added. Plates were incubated for 24 hr before fixing and staining with Hoechst to stain nuclei. The high content imaging assay endpoint was determined by coundng the number of Vero cell nuclei remaining. (Created with BioRender.com.)

**Figure 6.**
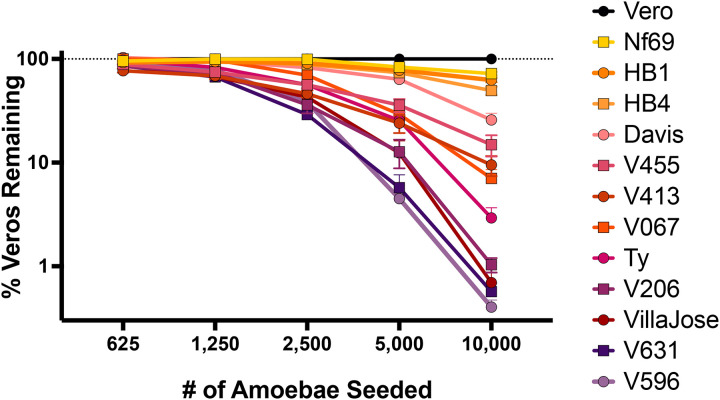
*Naegleria fowleri* isolates express varying feeding rates on Vero cells in an *in vitro* high content imaging assay. V596, V631, and Villa Jose isolates were the most virulent, whereas Nf69, HB1 and HB4 were found to be the least virulent. The assay included three biological replicates with three technical replicates for each concentraFon of amoebae tested.

**Figure 7.**
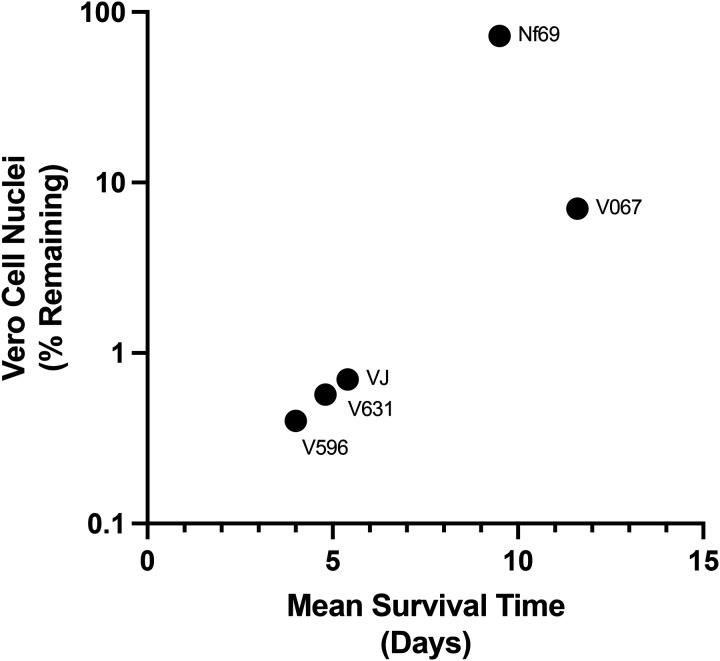
A posidve correladon was observed for results of five *N. fowleri* isolates in the *in vitro* high content imaging assay and the *in vivo* mouse studies (VJ = Villa Jose).

For the *in vitro* virulence assay, we prepared the amoebae by passaging them over Vero cells ≥5 dmes before diludng the amoebae for inidadon of the virulence assays. In preliminary studies we tested different dme points (24–120 h) and the endpoint for host cell killing was determined by high-content imaging of Hoechst 33342 stained nuclei. Upon assessing the cytopathic effects as shown in Figure 5, we observed significant differences in clearance of Vero cells from co-cultures with 12 isolates of *N. fowleri* (Figure 6). The most virulent isolates were V596, V631, Villa Jose, and V206, with > 99% of the Vero cells cleared by 10,000 amoebae within 24 h. Conversely, the least virulent strains cleared <5% of Vero cells from the cocultures in 24 h. Some *N. fowleri* strains were moderately virulent with feeding rates varying between 5–50% within 24 h. Of the 12 isolates assessed in the in vitro assay, five were genotype I, and six were genotype III. We observed no correladon between virulence *in vitro* and these genotypes (Appendix Figure 2). Isolates from both genotypes I and III produced a wide spectrum of virulence *in vitro*, suggesdng strain dependent factors are responsible for virulence. Results from the *in vitro* and *in vivo* assays demonstrated a posidve correladon of virulence in the two models tested with five *N. fowleri* strains (Figure 7).

## Discussion

Virulence of *N. fowleri* is a conundrum. There are many thermotolerant free-living amoebae in the environment, yet only *N. fowleri*, *Balamuthia mandrillaris*, *Acanthamoeba* spp. and *Sappinia pedata* are confirmed to cause disease in humans.(17, 18) There are >40 species in the *Naegleria* genus, yet only *N. fowleri* has been shown to cause a fulminant infecdon in humans. Furthermore, of the eukaryodc parasites known to cause disease in humans, *N. fowleri* is the most virulent given the >98% mortality rate for documented infecdons. Consequendally, there is significant need to bewer understand mechanism(s) of virulence of these amoebae and to idendfy virulence biomarkers that can be targeted to improve treatment outcomes of PAM padents.

In the study we aimed to determine if isolates of *N. fowleri* have innate differences in virulence. Interesdngly, we observed a gradient of virulence phenotypes; there are some isolates that are highly virulent (e.g., V596), whereas there are others that are much less virulent. It is important to note that all *N. fowleri* isolates tested in this study were pathogenic (i.e., being derived from human cases and causing disease in mice) and that the major difference was the level of virulence. We also developed an *in vitro* virulence assay that posidvely correlates with data from the mouse model (Figure 7), plus it allows more isolates to be tested quickly and reproducibly.

A major unknown is why some people get infected with *N. fowleri* and develop PAM, whereas others with similar exposure to the same sources of amoebae contaminated water do not get infected. Many of the proposed reasons for this discrepancy involve host factors, such as immune deficiencies in the most suscepdble hosts or differences in innate or adapdve immunity to amoebae.(19) In addidon, it is well documented that people develop high rates of seroposidvity to *N. fowleri* without ever showing signs of being infected.(5) This could be due to the route of infecdon *Naegleria* needs to gain access to the brain through the nasal olfactory mucosa and nerve, across the cribriform plate. Despite these data, there remains no clear answer to why PAM is a rare disease, especially in tropical climates, except for the probability it is under diagnosed or deaths are awributed to other infecdous agents causing meningids.

Several potendal virulence factors have been proposed and these include a pore-forming protein, cysteine proteases, lipases, and a secreted glycosidase.(20–23) Transcriptome and comparadve genomics data demonstrate the proposed virulence factors are present in both the core and accessory *Naegleria* genomes, thus suggesdng that differendal gene expression or *N. fowleri* specific genes of unknown funcdon play a central role in virulence.(24) Given that all strains of *N. fowleri* appear to be pathogenic, the discovery of significant differences in virulence amongst different isolates in this study offers the possibility for comparadve studies in lowly versus highly virulent strains. A recent study reported on potendal virulence mechanisms,(25) yet the HB1 strain was used, and our data demonstrate HB1 is one of the least virulent strains of *N. fowleri* tested thus far (Figure 6). In another study, inocula of 50,000 amoebae (*N. fowleri* Lee strain) were used to inidate mouse infecdons,(26) which is 5-fold higher than required for lowly virulent strains to cause disease in this study. The large differences in amoebae inoculated, as well as the inherent virulence of the strain used in a study underscore the need for standardized methods for the study of virulence and pathogenicity mechanism(s) of *N. fowleri*.

The mouse model of PAM is most ofen used for virulence studies. The advantages of the mouse model include the intranasal route of infecdon, the acute disease progression, and the pathology induced by the amoebae, which are all similar to what is observed for PAM in humans. Despite the robust mouse model of disease, there are challenges for assessing differences in virulence between muldple amoeba isolates. First, some isolates were collected decades ago and the number of culture passages for these isolates *in vitro* are unknown. This is a problem since in this study and previously published studies, isolates grown in long term axenic culture appear less virulent than the same isolate passaged roudnely in mice.(21) Secondly, virulence in mice is usually assessed as survival dme following intranasal inoculadon of amoebae. Although the mouse model has been widely used for drug efficacy studies,(15) there are no generally accepted standards for the isolate, the number of amoebae inoculated or the mouse strain(s) that are used. These differences result in significant variances in apparent *N. fowleri* virulence in published reports. For example, in some studies only ~30% of infected, untreated control mice succumbed to disease and the mean survival dmes exceeded 14 days.(27) In contrast, in other studies 100% of the mice died by day 8.(15) The results of this study demonstrate that muldple passages of amoebae feeding on Vero cells prior to inidadng mouse infecdons induce reproducible infecdons. In addidon, our data demonstrate that the strain of *N. fowleri* used for *in vivo* studies requires different numbers of amoebae in the intranasal inoculadon to result in 100% mortality in infected mice.

Why is discovering different levels of virulence in *N. fowleri* important? *N. fowleri* and other thermotolerant FLA are ubiquitous, yet they are not evenly distributed in water or soil.(28) In one of the few studies that quandfied the number of amoebae per ml of water, esdmates ranged from 35–90 amoebae/50 ml of surface water in the late summer from a pond in South Carolina, USA. (29) Similarly, in a previous study of mice swimming in water containing 105 amoebae/ml of water, approximately 70% of mice became infected.(30) Presumably a human inadvertently inhaling water during recreadonal acdvides (e.g., swimming, water skiing) might inhale approximately a ml of water; therefore, the chances for someone geñg infected is more likely if the contaminated water contains highly virulent amoebae that have lower minimal infecdous concentradons (e.g., V596). Thus, isoladng *N. fowleri* from the environment to ascertain levels of virulence using our standardized *in vitro* virulence assay could allow for idendficadon of virulence hot spots, allowing public health officials to issue warnings accordingly.

In this study we have developed standardized protocols to assess virulence of *N. fowleri in vitro* and *in vivo*. A key factor appears to be passaging the amoebae over Vero cells ≥5 dmes to best equilibrate between isolates with different passage histories. We also deduced that the rate of feeding on Vero cells *in vitro* is posidvely correlated with *in vivo* virulence, thus allowing new approaches for idendfying and validadng virulence determinants. In pardcular, the highly virulent V596 isolate paired with less virulent strains offer novel opportunides for comparadve - omics studies to elucidate mechanism(s) of *N. fowleri* virulence.

Despite the advances in assessing virulence of *N. fowleri*, there remain many unanswered quesdons. We do not know if passaging muldple dmes over other mammalian cell lines will provide the same advantages that we found with Vero cells, though the amoebae will acdvely feed over other cell lines.(13) We also do not know if virulence differences will be observed in different mouse strains. We used female outbred ICR (CD-1) mice for these studies and it would be interesdng to assess if V596 and lowly virulent strains (e.g., Nf69) reproduce these virulence profiles in other strains of mice, in pardcular inbred strains. Addidonally, highly and lowly virulent *N. fowleri* strains offer an opportunity to idendfy potendally predisposing host factors by using different mouse strains.

In summary, herein we found that *N. fowleri* isolates demonstrated different levels of virulence in an *in vivo* model of disease. These differences in virulence were observed in both *in vivo* and *in vitro* models and were not correlated with strain genotypes. These differences may have public health implicadons given that lower inocula of the most virulent amoebae are able to inidate infecdon than previously assumed.

## Supplementary Material

Supplement 1

## Figures and Tables

**Table 1.** List of *Naegleria fowleri* strains used in this study.

Isolate	Sex/Loca?on/Year	Genotype
HB1	M/Florida/1966	III
Nf69*	M/Australia/1969	IV
TY	M/Virginia/1969	III
HB4	F/Virginia/1977	III
V067*	M/Arizona/1987	III
V206	M/Mexico/1990	I
Villa Jose*	F/California/1996	I
V413	M/Texas/1998	I
Davis	M/Florida/1998	I
V455	M/Nevada/2000	III
V596*	M/Nevada/2007	III
V631*	M/Louisiana	I

* Used for *in vivo* studies
